# Therapeutic potential of Lianhua Qingke in airway mucus hypersecretion of acute exacerbation of chronic obstructive pulmonary disease

**DOI:** 10.1186/s13020-023-00851-4

**Published:** 2023-11-03

**Authors:** Yuanjie Hao, Tongxing Wang, Yunlong Hou, Xiaoqi Wang, Yujie Yin, Yi Liu, Ningxin Han, Yan Ma, Zhen Li, Yaru Wei, Wei Feng, Zhenhua Jia, Hui Qi

**Affiliations:** 1 Graduate School, Hebei Medical University, Shijiazhuang, 050017 Hebei China; 2 Hebei Academy of Integrated Traditional Chinese and Western Medicine, Shijiazhuang, 050035 Hebei China; 3 National Key Laboratory for Innovation and Transformation of Luobing Theory, Shijiazhuang, 050035 China; 4 Graduate School, Hebei University of Chinese Medicine, Shijiazhuan, 050090 Hebei China; 5 Affiliated Yiling Hospital of Hebei Medical University, Shijiazhuang, 050091 Hebei China

**Keywords:** Lianhua Qingke (LHQK), AECOPD, Airway inflammation, Goblet cell hyperplasia, Muc5ac, AQP5, Club cells

## Abstract

**Background:**

Lianhua Qingke (LHQK) is an effective traditional Chinese medicine used for treating acute tracheobronchitis. In this study, we evaluated the effectiveness of LHQK in managing airway mucus hypersecretion in the acute exacerbation of chronic obstructive pulmonary disease (AECOPD).

**Methods:**

The AECOPD model was established by subjecting male Wistar rats to 12 weeks of cigarette smoke (CS) exposure (80 cigarettes/day, 5 days/week for 12 weeks) and intratracheal lipopolysaccharide (LPS) exposure (200 μg, on days 1, 14, and 84). The rats were divided into six groups: control (room air exposure), model (CS + LPS exposure), LHQK (LHQK-L, LHQK-M, and LHQK-H), and a positive control group (Ambroxol). H&E staining, and AB-PAS staining were used to evaluate lung tissue pathology, inflammatory responses, and goblet cell hyperplasia. RT-qPCR, immunohistochemistry, immunofluorescence and ELISA were utilized to analyze the transcription, expression and secretion of proteins related to mucus production in vivo and in the human airway epithelial cell line NCI-H292 in vitro. To predict and screen the active ingredients of LHQK, network pharmacology analysis and NF-κB reporter system analysis were employed.

**Results:**

LHQK treatment could ameliorate AECOPD-triggered pulmonary structure damage, inflammatory cell infiltration, and pro-inflammatory cytokine production. AB-PAS and immunofluorescence staining with CCSP and Muc5ac antibodies showed that LHQK reduced goblet cell hyperplasia, probably by inhibiting the transdifferentiation of Club cells into goblet cells. RT-qPCR and immunohistochemistry of Muc5ac and APQ5 showed that LHQK modulated mucus homeostasis by suppressing Muc5ac transcription and hypersecretion in vivo and in vitro, and maintaining the balance between Muc5ac and AQP5 expression. Network pharmacology analysis and NF-κB luciferase reporter system analysis provided insights into the active ingredients of LHQK that may help control airway mucus hypersecretion and regulate inflammation.

**Conclusion:**

LHQK demonstrated therapeutic effects in AECOPD by reducing inflammation, suppressing goblet cell hyperplasia, preventing Club cell transdifferentiation, reducing Muc5ac hypersecretion, and modulating airway mucus homeostasis. These findings support the clinical use of LHQK as a potential treatment for AECOPD.

**Supplementary Information:**

The online version contains supplementary material available at 10.1186/s13020-023-00851-4.

## Introduction

Acute exacerbations of chronic obstructive pulmonary disease (AECOPD) are characterized by the sudden worsening of chronic obstructive pulmonary disease (COPD) symptoms such as increased dyspnea, cough, sputum production, and purulence [1] and may be accompanied by tachypnea and/or tachycardia [2]. These events often result in increased local and systemic inflammation and a decline in lung function, leading to the substantial morbidity and mortality [3, 4]. Current pharmacological approaches to reduce the risk of future exacerbations include treatment with long-acting bronchodilators, inhaled steroids, mucolytics, vaccinations, and long-term macrolides. However, these approaches do not prevent the progression of the disease [5].

Typically, AECOPD is triggered by bacterial or viral infections and non-infectious stimuli, such as air pollution. Mechanistically, risk factors may activate the transcription factor nuclear factor (NF)-κB in airway epithelial cells and macrophages [6], which then release inflammatory cytokines, including interleukin (IL)-8, tumor necrosis factor-alpha (TNF-α), IL-6, and chemokines such as CCL5 and CLCL5 [7, 8]. These molecules attract T lymphocytes (CD3), eosinophils, and neutrophils [9]. In addition, the elevated expression of proteases, such as matrix metallopeptidase 9 (MMP-9), which may destroy the lung structure, was independently associated with COPD exacerbations [10]. Subsequently, elevated inflammation may result in secondary goblet cell hyperplasia and mucus hypersecretion, which can lead to airway obstruction, impairment of the pathogen and respiratory hazard clearance [11], airway inflammation, and deterioration of lung function [12]. This vicious cycle of infection and injury can worsen COPD, making inflammation and mucus hypersecretion not only symptoms but also risk factors of AECOPD [13]. Therefore, drugs targeting both inflammation and excessive mucus production to effectively alleviate airway obstruction are critical for AECOPD interventions.

Goblet cell hyperplasia is a characteristic feature of COPD and contributes to pathological mucus hypersecretion [14, 15]. Experimental evidence has confirmed that goblet cells may be derived from Club cells (also known as Clara cells), which are major airway epithelial cells characterized by Club cell secretory protein (CCSP) secretion and contribute to airway epithelial cell regeneration after lung injury [16]. The transdifferentiation of Club cells to goblet cells has been observed in pulmonary diseases characterized by goblet cell hyperplasia, including asthma, COPD, and respiratory infection disease [17]. Transdifferentiated Club cells can be identified by low CCSP expression tending toward a CCSP/TFF1 [18] or a CCSP/Muc5ac [17] double-positive status. Multiple molecular pathways, including epidermal growth factor receptor (EGFR), interleukin (Th2 cytokines), Notch signal transducer and activator of transcription 6 (STAT 6), and WNT signaling pathways [19], are reportedly involved in goblet cell differentiation.

Mucins, synthesized primarily by goblet cells and mucous cells of the submucosal glands, are the key protein components of airway mucus. Muc5ac is a dominant gel-forming mucin expressed and secreted by airway goblet cells [20]. In addition, water constitutes 95% of mucus found in airways. This makes liquid homeostasis an important determinant of the quantity and quality of mucus [21]. Aquaporin 5 (AQP5), a member of the AQP family of proteins, plays a crucial role in pulmonary airway water transport and regulates the proportion of fluid in airways [22]. Reduced water levels in mucus can lead to increased viscosity, impaired airway clearance, and a greater risk of bacterial infection. This triggers inflammation and mucus hypersecretion [11, 23].

Cigarette smoking (CS) has also been identified as a risk factor for airway inflammation, goblet cell hyperplasia, excessive mucus secretion, and AECOPD [24]. Lipopolysaccharide (LPS) was shown to induce COPD in a short-term disease model [25]. When CS and LPS are administered concomitantly, it can induce AECOPD in rats [26], which typically manifests as mucus hypersecretion. In the present study, a rat AECOPD model was induced with cigarette smoke exposure and LPS instillation.

LHQK is a traditional Chinese medicine (TCM) composed of 15 herbs, including Mahuang, Shigao, Lianqiao, and Huangqin, among others. It is known for its ability to dispel phlegm, relieve cough, and improve lung ventilation [27]. Its efficacy in treating acute tracheitis and bronchitis, particularly in reducing cough and sputum, has been confirmed through its anti-inflammatory and anti-tussive properties and its ability to reduce sputum viscosity and promote drainage [28]. Evidence from recent studies has also confirmed its effectiveness in treating HCoV-229E-induced acute bronchitis and pneumonia [29, 30]. However, its impact on AECOPD remains unknown. Our findings show that LHQK effectively ameliorated excessive mucus accumulation in a rat AECOPD model by reducing pro-inflammatory cytokine production, preventing goblet cell hyperplasia, and balancing the airway mucus homeostasis.

## Materials and methods

### Animals

All animal experiments conducted in this study were carried out in strict accordance with the guidelines and approval of Experimental Animal Ethical Committee of Hebei Yiling Pharmaceutical Research Institute (No. N2021162). Six-week-old Male Wistar rats (180–220 g) were purchased from Beijing Weitong Lihua Experimental Animal Technology Co., Ltd. (Beijing, China). These rats were randomly divided into six groups (n = 10 per group), including control group (room air exposure), model group (CS + LPS exposure), LHQK low dose group (LHQK-L), LHQK middle dose group (LHQK-M), LHQK high dose group (LHQK-H) and Ambroxol group (a positive control group). Rats in the control and CS + LPS groups were perfused with 0.5% carboxymethylcellulose (CMC) sodium solution, whereas rats in the LHQK-L, LHQK-M, and LHQK-H groups received an intragastric administration of LHQK at doses of 1.47, 2.93, and 5.86 g/kg/d, respectively. The Ambroxol group received intragastric administration of Ambroxol at a dose of 30 mg/kg. Both of LHQK and Ambroxol were administered daily for a period of 12 weeks, starting from the first day of cigarette smoke (CS) exposure.

To establish the AECOPD model, the rats were exposed to CS (80 cigarettes/day for 5 days per week) and intratracheal instillation of LPS (LPS, Sigma, USA) instillation. Commercial cigarettes were produced by Shanghai Tobacco Industry Co., Ltd (Hongshuangxi Filter Cigarette, Shanghai, China). 200 μg LPS (prepared as 1 mg/mL solution in saline) was instilled intratracheally on day 1, day 14, and 24 h before sample collection. The instillation procedure was performed under anesthesia using 5% isoflurane.

### Reagents preparation

Lianhua Qingke (LHQK, Lot No. 2104001) was provided by Shijiazhuang Yiling Pharmaceutical Co., Ltd. (Shijiazhuang, China). LHQK is a patented TCM composed of *Ephedra sinica* Stapf, *Forsythia suspensa* (Thunb.) Vahl, *Scutellaria baicalensis* Georgi, *Morus Alba* L., *Prunus sibirica* L., *Peucedanum praeruptorum* Dunn, *Pinellia ternate* (Thunb.) Breit., *Citrus reticulata* Blanco, *Fritillaria thunbergii* Miq., *Arctium lappa* L., *Lonicera hypoglauca* Miq., *Rheum palmatum* L., *Platycodon grandiflorus* (Jacq.) A. DC., *Glycyrrhiza uralensis* Fisch., and Gypsum fibrosum. To prepare the LHQK solution for animal experimental use, the LHQK tablets were dissolved in a 0.5% carboxymethylcellulose (CMC) sodium solution at concentrations of 0.147 g/mL, 0.293 g/mL, and 0.586 g/mL. Additionally, for in vitro cellular experimental use, the LHQK tablets were dissolved in dimethylsulfoxide (DMSO), subjected to stirring for a duration of one hour, followed by sonication for an additional hour. Subsequently, the mixture was centrifuged at 8000 rpm for 30 min. The supernatant was subsequently filtered using a 0.22 µm pore size filter. The non-toxic concentrations of 125 µg/mL and 250 µg/mL were used for the in vitro experiments.

### Hematoxylin and eosin (H & E) staining

Lung tissues were dissected after perfusion and immediately fixed in a 4% paraformaldehyde solution. They were then dehydrated in gradient alcohol solutions, embedded in paraffin, sectioned, and stained with hematoxylin and eosin (H&E) (Beyotime, Shanghai, China). Finally, the slices were photographed under a fully automated biological microscope (Leica DM6000B, Wetzlar, Germany). Mean linear intercept (MLI) and mean alveolar number (MAN) were calculated according to the literatures [31].

### Alcian blue-periodic acid-Schiff (AB-PAS) staining

Alcian Blue/Periodic Acid-Schiff (AB-PAS) staining was performed with lung paraffin sections to detect goblet cell metaplasia of bronchial epithelium. Images were captured by Leica light microscope (Leica DM6000B, Wetzlar, Germany). The AB-PAS-positive area and total area of corresponding bronchial epithelium were measured by Image-Pro Plus 6.0. Data were presented as the ratio of AB-PAS-positive area to the total area.

### Immunohistochemistry (IHC)

Tissue paraffin samples were cut into 3 μm sections. Thees sections were first deparaffinized in 1 mM ethylenediaminetetraacetic acid (EDTA) at 95 °C for 20 min, and then incubated with 3% hydrogen peroxide for 20 min. Subsequently, the sections were blocked using 10% goat serum for 90 min at room temperature. Primary antibodies against Muc5ac (Abcam, ab3649, 1:2000) or AQP5 (Abcam, ab78486, 1:2000) were applied, and the sections were left to incubate overnight at 4 °C. Subsequently, the sections were washed with PBS and incubated with a second antibody kit (Zhongshanjinqiao, Beijing, China) for 30 min at 37 °C. After rinsing in PBS, the sections were developed with 3,3′—diaminobenzidine (DAB). Finally, images were captured using a Leica light microscope. Immunohistochemical staining results were evaluated and quantified using ImageJ (Version 1.53).

### Cell culture and cellular model for mucus hypersecretion

The human airway epithelial cell line NCI-H292 (CL-0167, Procell, China) were cultured in RPMI Medium1640 (Thermo Fisher Scientific, USA) supplemented with 10% FBS, penicillin (100 U/mL) and streptomycin (100 µg/mL), and incubated at 37 °C in a 5% CO_2_ humidified atmosphere. The cells were randomly divided into 4 groups: the control group, the cigarette smoke extract and LPS expsure group (CSE + LPS), the LHQK low-dose group (LHQK-L) and the LHQK high-dose group (LHQK-H). The control group was cultured under normal conditions, the CSE + LPS group was incubated with 50 µg/mL CSE (AAPR551-2, PythonBio, China) in combination with 10 μg/mL LPS (Sigma, USA) to establish the cell model of airway mucus hypersecretion for 24 h. The LHQK-L group and the LHQK-H group were incubated with 125 µg/mL and 250 µg/mL of LHQK together with CSE + LPS exposure.

### Immunofluorescence (IF) staining and quantification

Lung paraffin sections were deparaffinized and subjected to antigen retrieval, and then blocked with 3% hydrogen peroxide for 10 min at 37 °C in the dark. Subsequently, the sections were blocked with 5% bovine serum albumin (BSA) for 30 min at room temperature before incubated with primary antibodies against CD45 (Santa Cruz Biotechnology, sc-19597, 1:200), CD68 (Abcam, ab125212,1:500), CD3 (Proteintech, 17,617-1-AP, 1:200), CCSP (Abcam, ab213203, 1:100) and Muc5ac (Abcam, ab3649, 1:200) at 4 °C overnight. The next day, the sections were washed with PBS and incubated with the secondary antibodies of goat anti-rabbit IgG H&L (Alexa Fluor^®^ 488, Abcam, ab150081,1:500), goat anti-mouse IgG (H + L) (CoraLite594, Proteintech, SA00013-3, 1:500), Goat Anti-Rat IgG H and L (Alexa Fluor^®^ 555, Abcam, ab150158, 1:500) and Donkey Anti-Rabbit IgG H and L (Alexa Fluor^®^ 647, Abcam, ab150075, 1:500), respectively, for 50 min at room temperature in the dark before being stained with 4′, 6-diamidino-2-phenylindole (DAPI) (Solarbio, Beijing, China). For the IF of Muc5ac in NCI-H292 cells, the cells were fixed with 4% paraformaldehyde for 15 min followed by permeabilization using 0.25% Triton X-100 for 5 min. Subsequently, the cells were blocked with 1% BSA for 1 h at room temperature before being subjected to Muc5ac IF staining. The sections were observed under a Zeiss confocal microscope (Oberkochen, Germany). The antibodies used in the experiment are shown in Table 1. The quantitative analysis of the ratio for CCSP^+^Muc5ac^+^/CCSP^+^ was performed in five fields selected randomly from each stained sections. In each field, the number of cells with CCSP^+^ signal (Green) and the number of cells with CCSP^+^ and Muc5ac^+^ double-positive signals (Yellow) were counted, respectively. Subsequently, the ratio of double positive cell numbers within the CCSP + cell population was computed to determine the relative extent of Club cell transdifferentiation into goblet cells. The mean immunofluorescent intensity of CCSP and Muc5ac was evaluated and quantified using the software ImageJ (Version 1.53).

**Table 1:** Primary and secondary antibodies

Antibodies	Company	Dilution
Anti-CD45	Santa Cruz Biotechnology, sc-19597	1:200
Anti-CD68	Abcam, ab125212	1:500
Anti-CD3	Proteintech, 17,617-1-AP	1:200
Anti-CCSP	Abcam, ab213203	1:100
Anti-Muc5ac	Abcam, ab3649	1:100
Goat anti-rabbit IgG H&L	Abcam, ab150081	1:500
goat anti-mouse IgG H&L	Proteintech, SA00013-3	1:500
Goat anti-rat IgG H&L	Abcam, ab150158	1:500
Donkey anti-rabbit IgG H&L	Abcam, ab150075	1:500
Goat anti-mouse IgG H&L	Proteintech, SA00013-1	1:500

### Real-time quantitative polymerase chain reaction (RT-qPCR)

Total RNA was extracted from the rat lungs or NCI-H292 cells using Trizol-Reagent (Invitrogen, USA). Reverse transcription was performed using Prime Script™ RT Reagent Kit (Takara Bio, Japan) to obtain cDNA, and RT-qPCR was performed using SYBR Green PCR Master Mix (Takara Bio, Japan) according to the manufacturer’s instructions. The PCRs were performed and recorded on the QuantStudio RT-qPCR system (Thermo Fisher Scientific, Massachusetts, USA). The relative mRNA expression of target genes in each sample was calculated using the relative quantitative formula 2^–ΔΔCt^, where ΔCt value = target gene Ct value—GAPDH gene Ct value [32]. GAPDH was used as the internal reference gene. The primers of Muc5ac, AQP5, and GAPDH were synthesized by Dingguo Biotech Co., Ltd. (Beijing, China) and the primer sequences were shown in Table 2.

**Table 2:** Primers sequence used for RT-qPCR

Species	Gene	Primer Sequence (5′ — 3′ )
Rat	GAPDH	F:5′—CTGGAGAAACCTGCCAAGTATG—3′
Rat		R:5′—GGTGGAAGAATGGGAGTTGCT—3′
Rat	Muc5ac	F:5′—AACTCTGCCCACCACAAGC—3′
Rat		R:5′—TGCCATCTATCCAATCAGTCCAAT—3′
Rat	AQP5	F:5′—ACCATGAAAAAGGAGGTGTGCT—3′
Rat		R:5′—TTGAGATTTGCAGAATGGTGGG—3′
Human	GAPDH	F:5′—AGAAGGCTGGGGCTCATTTG—3′
Human		R:5′—AGGGGCCATCCACAGTCTTC—3′
Human	Muc5ac	F:5′—CAGCACAACCCCTGTTTCAAA—3′
Human		R:5′—GCGCACAGAGGATGACAGT—3′

*F* forward, *R* reverse

### ELISA

Five milliliters of bronchoalveolar lavage fluid (BALF) was collected from the left lobe of the lung and then centrifuged at 3000 rpm for 10 min at 4 °C. Supernatant of BALF was used to measure the protein levels of TNF-α (Proteintech, KE20001), IL-1β (Proteintech, KE20005), IL-13 (Elabscience, E-EL-R0563c), MMP-9 (Proteintech, KE20006) using ELISA kits following the manufacturer's instructions.

### Network pharmacology analysis

Here, we combined network pharmacology technology in combination bioinformatics analysis to investigate the effects of LHQK on airway mucus hypersecretion. Genes closely related to the airway mucus hypersecretion were collected from GeneCards v5.16.0 [33] and CTD database revision 17071 M [34]. The gene symbols were normalized by UniProt [35]. Fourteen components identified from LHQK tablets in Wang, M., et al. [29], including neochlorogenic acid, chlorogenic acid, cryptochlorogenic acid, isoforsythiaside, phillygenin, hesperidin, baicalin, arctiin, aloe-emodin, glycyrrhizic acid ammonium salt, rhein, emodin, 1,8-dihydroxy-3-methylanthraquinone, and physcion were used for subsequent analysis. To improve reliability, the comprehensive target spectrum of 14 ingredients was obtained by combing the known targets collected from DrugBank [36], TTD [37], ChEMBL [38], PubChem [39], and CTD [34], and the putative targets predicted from STITCH [40], SEA [41], TargetNet [42], SwissTargetPrediction [43], ChEMBL_prediction [44], and BATMAN-TCM [45] (as shown in Table 3). The compound-target (C-T) network was constructed and visualized by Cytoscape v3.7.1 [46]. The degree computed by NetworkAnalyzer plugin [47] was used to evaluate the importance of the nodes. STRING database version 11.5 (https://cn.string-db.org/) [48] was used to build the protein–protein interaction (PPI) network (confidence = 0.4). ClueGO plugin in Cytoscape was used to decipher functionally grouped biological process (BP) enrichment analysis [49]. The mechanism targets for the intervention effect of LHQK on airway mucus hypersecretion were obtained by intersection analysis between the airway mucus hypersecretion genes and targets of LHQK. Subsequently, Metascape (https://metascape.org/gp/index.html) was adopted to perform functional enrichment analysis [50].

**Table 3:** 11 sources for obtaining target profiles of LHQK ingredients

#	Source category	Database	Interaction type	Selection criteria
1	Approved drug database	DrugBank	Known	All
2		TTD database	Known	All
3	Activity assay database	ChEMBL	Known	All
4		PubChem	Known	All
5	Literature mining database	STITCH	Text-mining	Score ≥ 0.9
6		CTD	Text-mining	All
7	Target prediction tool	TargetNet	Putative	Score ≥ 0.85
8		SwissTargetPrediction	Putative	Score ≥ 0.9
9		ChEMBL prediction tool	Putative	Active under 90% confidence
10		BATMAN-TCM	Putative	Score ≥ 0.48
11		SEA	Putative	*P* < 10^–16^ and Max Tc ≥ 0.3

### Luciferase reporter assay

The GloResponse™ NF-κB-RE-luc2P HEK293 cell line (Promega, Cat. # E8520, USA) was used to screen the effect of LHQK and its active components [29] on TNF-α (Biovision; 10 ng/mL) mediated nuclear translocation of NF-κB according to manufactures instruction. Cells were cultured in DMEM media with 10% fetal bovine serum and 1 × antibiotic–antimycotic (Gibco, Life Technologies) at 37 °C in a humidified incubator under 5% carbon dioxide. MTS and cytotoxicity assay kit (Sigma Aldrich) was used to assess cell viability, and the maxium non-toxic concentration for each active ingredient tested in Additional file 1: Figure S1 was selected as the dosage for the NF-kB luciferase reporter assay. Luciferase reporter assay was performed as reoprted. Briefly, cells were seeded in a white glass-bottom 96-well plate (Thermo Scientific), and pre-treated with active ingredients of LHQK for 2 h before TNF-α was added to trigger NF-κB activation. Luciferase activity was analyzed with ONE-Glo™ luciferase assay reagent using GloMax multimode reader (TECAN, Switerland).

### Statistical analysis

Statistical analysis was performed using SPSS 22.0 software (IBM; Armonk, NY, USA). One-way analysis of variance (ANOVA) was performed for multiple comparisons. *P* < 0.05 was defined as the threshold for statistical significance. The results were presented as the mean ± standard deviation (SD). Statistical graph generation was performed using GraphPad Prism (version 9.2.0).

## Results

### LHQK ameliorated lung injury and inflammatory cell infiltration

**Fig. 1:**
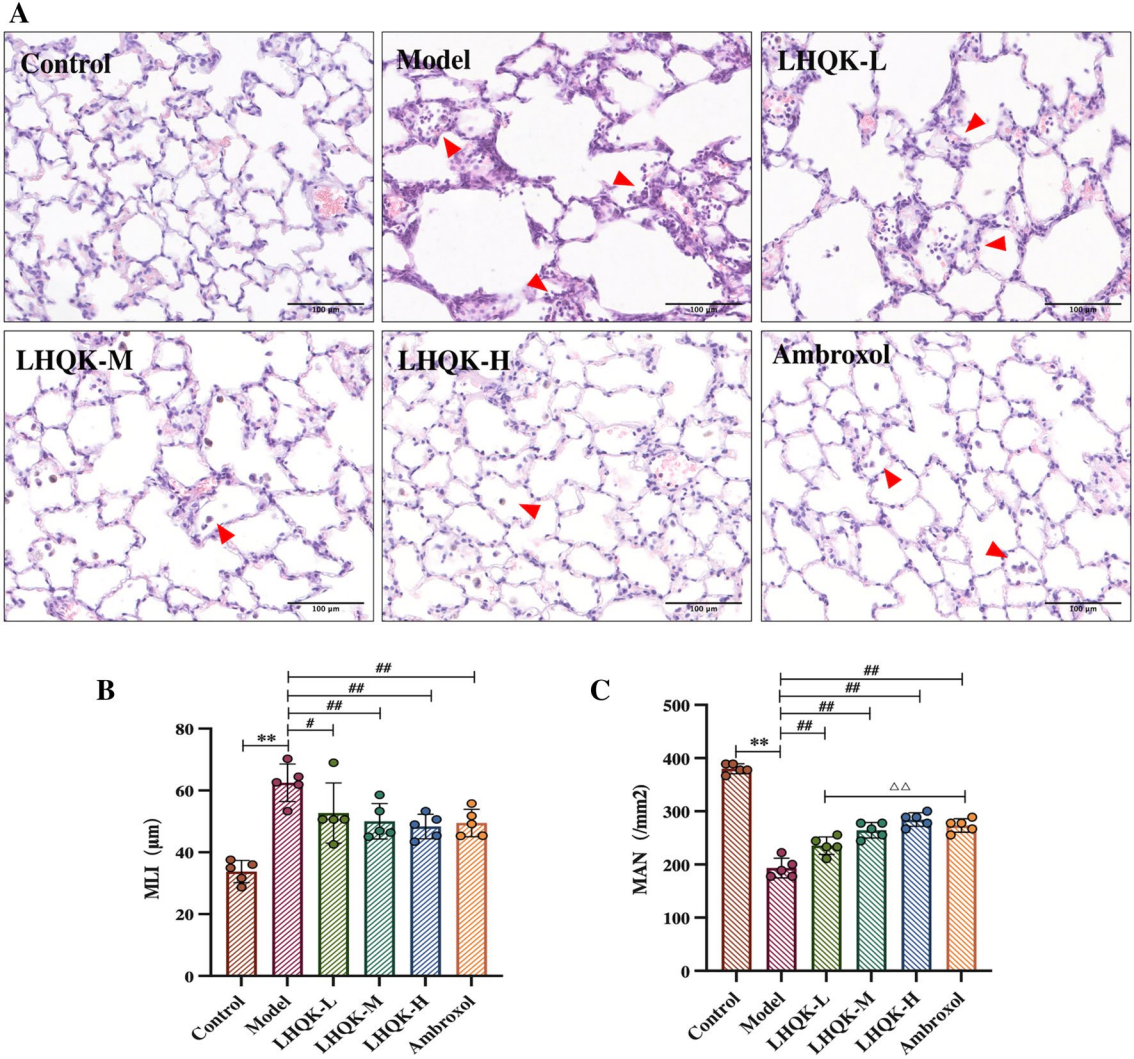
Morphological analysis and alveoli size and number in the lungs. **A** Pathological changes in the lungs (H&E staining magnification × 200) Bar: 100 μm. **B** Statistics of the mean linear intercept (MLI). **C** Statistics of the mean alveolar number (MAN). Data are presented as mean ± SD. **P < 0.01 vs. the control group; ^#^*P* < 0.05, ^##^*P* < 0.01 vs. the model group; ^∆∆^*P* < 0.01 vs. the Ambroxol group

Pathological changes in the lungs were assessed by H&E staining, and the alveolar size and alveolar number were quantified using MLI and MAN, respectively. Histological injuries, such as alveolar cavity dilation, alveolar wall breakage, airway thickening, alveolar septum widening, increases alveolar size, reduced alveolar number, and increased inflammatory cells infiltration, were observed in the model group (Fig. 1A). However, upon LHQK and Ambroxol (positive control) treatment, inflammatory cell infiltration was reduced, alveolar size was significantly decreased (LHQK-L: *P* < 0.05; LHQK-M, LHQK-H, and Ambroxol: *P* < 0.01) (Fig. 1B), and the alveolar number was increased (*P* < 0.01) (Fig. 1C). The alveolar number in the Ambroxol group was significantly higher than that in LHQK-L group (*P* < 0.01) but had no significant differences compared with that in the LHQK-M or LHQK-H group (*P* > 0.05) (Fig. 1C).

**Fig. 2:**
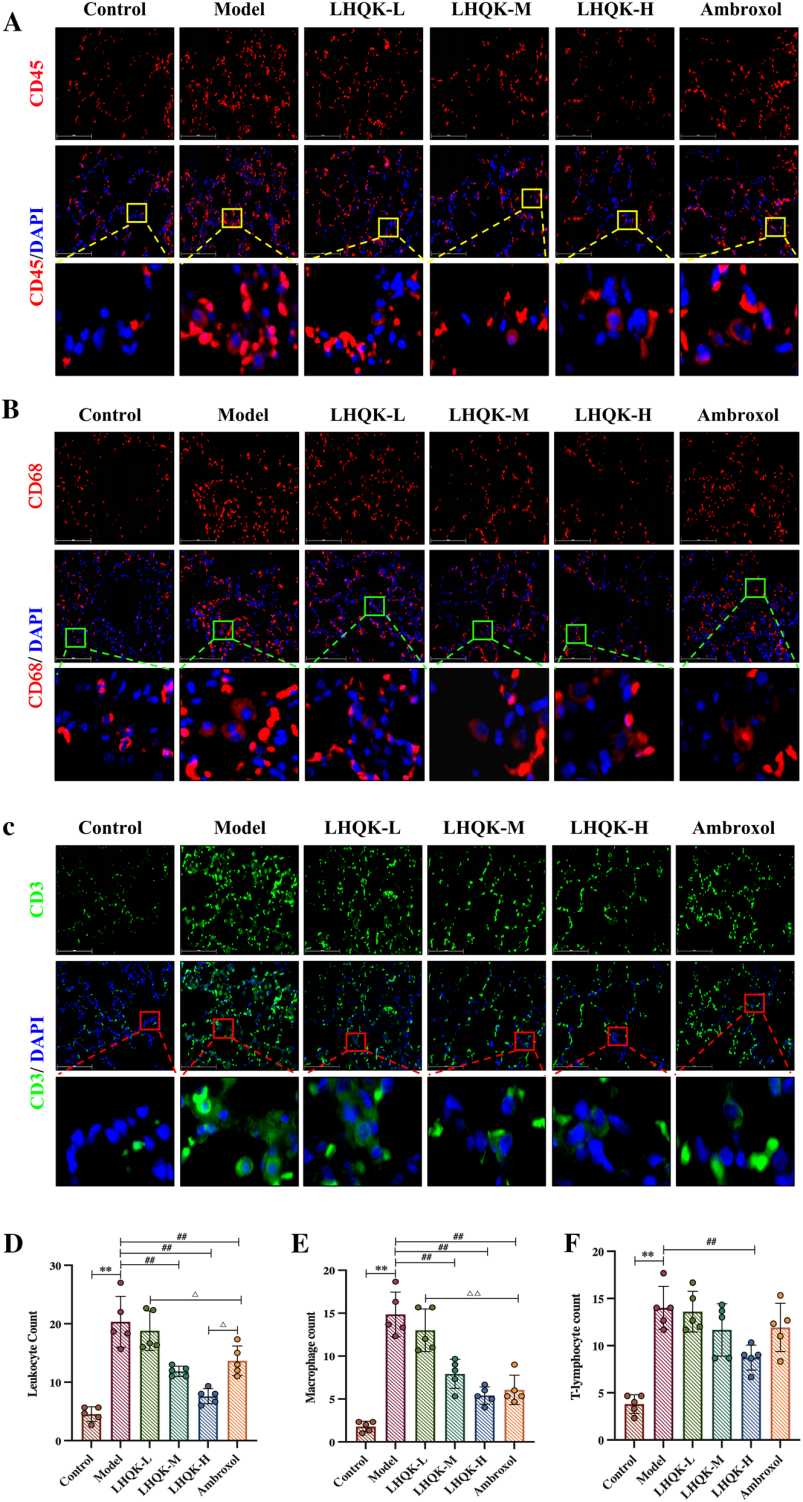
Effect of LHQK on inflammatory cell infiltration. **A–C** Immunofluorescence staining with CD45 (A), CD68 **B**, and CD3 **C** antibodies in lung tissues from rats (magnification × 400) Bar: 100 μm. **D–F** Quantification of leukocytes (CD45^+^), macrophages (CD68^+^), and T lymphocytes (CD3^+^) in rat lung tissues (n = 5). The values are expressed as mean ± SD. One-way ANOVA was used for statistical analysis. **P < 0.01 vs. the control group; ^##^*P* < 0.01 vs. the model group; ^∆^*P* < 0.05, ^∆∆^*P* < 0.01 vs. the Ambroxol group

To monitor the infiltrating inflammatory cells, the immunofluorescence staining was performed with markers for different inflammatory cells, including leukocytes (CD45^+^), macrophages (CD68^+^), and T lymphocytes (CD3^+^). The staining and quantification results showed that compared with that in the normal group, the number of leukocytes, macrophages, and T lymphocytes in the model group was significantly greater (*P* < 0.01) (Fig. 2A–F). Compared with that in the model group, the number of leukocytes and macrophages in the LHQK-M, LHQK-H, and Ambroxol groups and the number of T lymphocytes in the LHQK-H group were significantly lower (*P* < 0.01). The number of leukocytes in the Ambroxol group was significantly lower than that in the LHQK-L group (*P* < 0.05) but was significantly higher than that in the LHQK-H group (*P* < 0.05). The number of macrophages in the Ambroxol group was significantly lower than that in the LHQK-L group (*P* < 0.01).

**Fig. 3:**
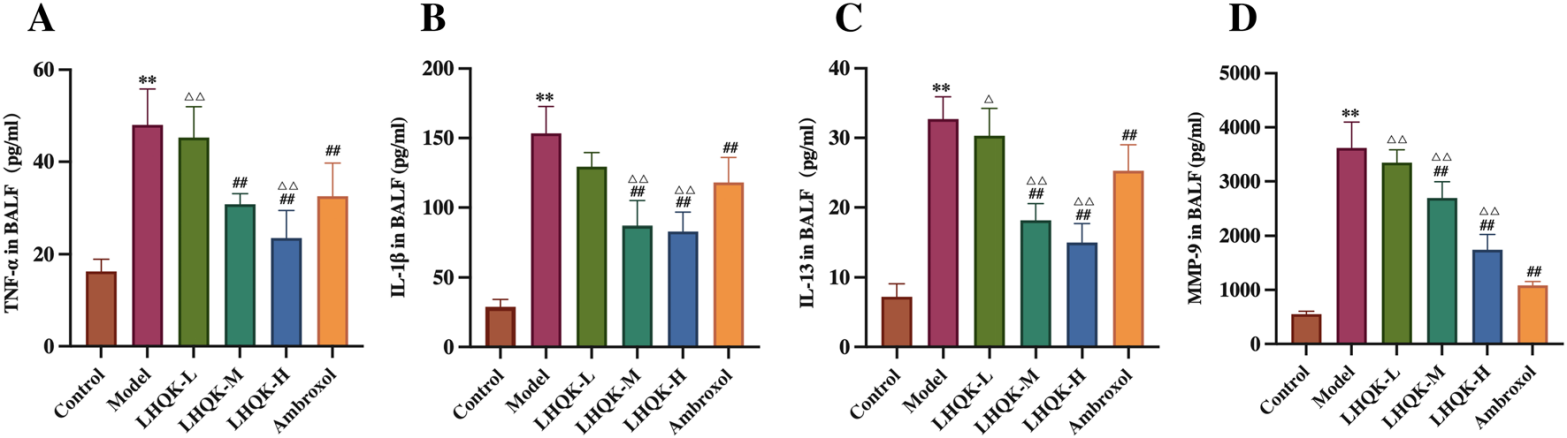
Effect of LHQK on the expression of pro-inflammatory cytokines, including TNF-α **A**, IL-1β **B**, IL-13 **C**, and MMP-9 **D** in BALF. The values are expressed as mean ± SD. One-way ANOVA was used for statistical analysis. **P < 0.01 vs. the control group; ^##^*P* < 0.01 vs. the model group; ^∆^*P* < 0.05, ^∆∆^*P* < 0.01 vs. the Ambroxol group

Next, proinflammatory cytokines, including TNF-α (Fig. 3A), IL-1β (Fig. 3B), IL-13 (Fig. 3C), and MMP-9 (Fig. 3D) in BALF were analyzed using ELISA. The levels of TNF-α, IL-1β, IL-13, and MMP-9 were significantly higher in the model group compared to the control group (*P* < 0.01) and significantly lower in the LHQK-M, LHQK-H, and Ambroxol groups (*P* < 0.01). Compared with those in the Ambroxol group, the levels of TNF-α, IL-1β, and IL-13 were significantly lower in the LHQK-H group (*P* < 0.01), but there was no significant difference in the level of MMP-9. Collectively, these data indicated that LHQK effectively ameliorated AECOPD-induced lung injury and inflammation phenotype.

### LHQK ameliorated goblet cell hyperplasia and the transdifferentiation of club cells to goblet cells

**Fig. 4:**
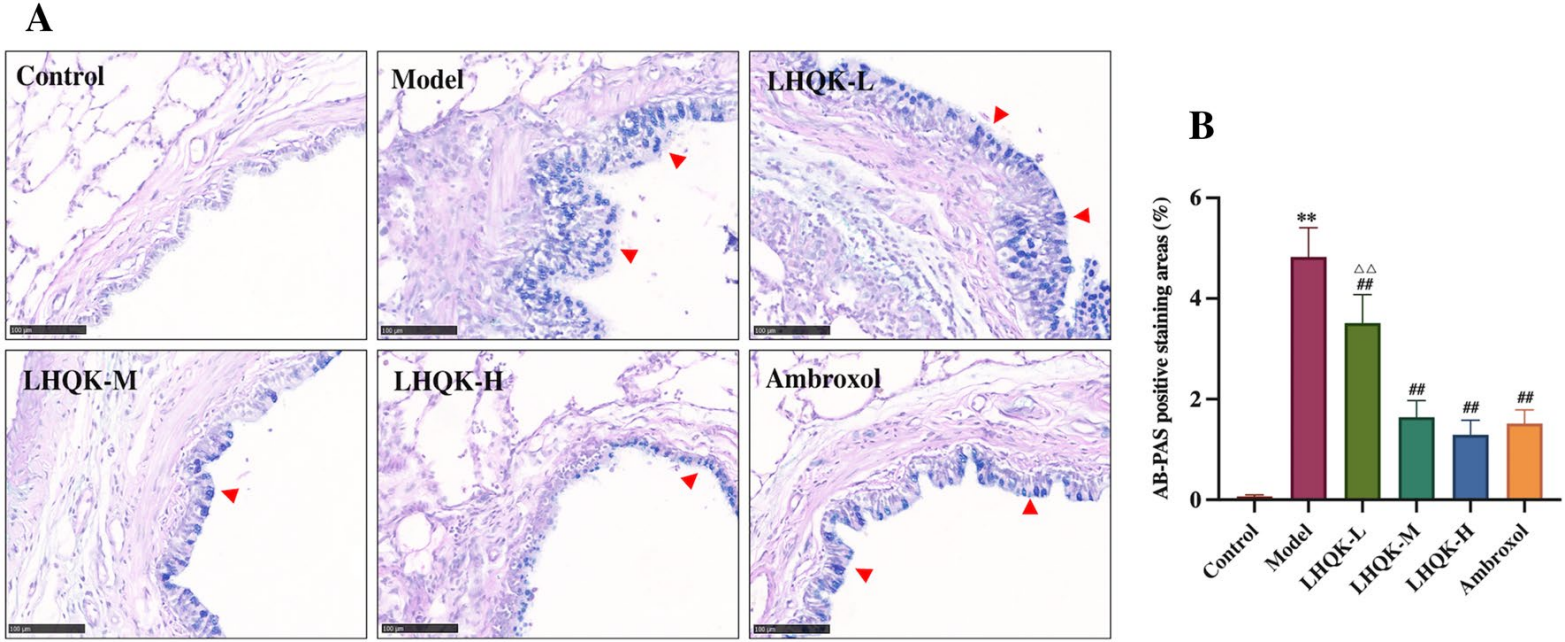
Effect of LHQK on goblet cell hyperplasia. **A** Goblet cells in the bronchial epithelium detected by AB-PAS staining (magnification × 200) Bar: 100 μm. The red arrowheads indicate the PAS-positive stains, showing blue or purple coloration. **B** Quantification of the PAS-positive staining area (%) in the airway (n = 5). AB-PAS-positive rates were determined based on the ratio of the AB-PAS-positive area to the total bronchiolar epithelial area. The values are expressed as mean ± SD. One-way ANOVA was used for statistical analysis. ^**^P < 0.01 vs. the control group; ^##^*P* < 0.01 vs. the model group; ^∆∆^*P* < 0.01 vs. the Ambroxol group

Given that inflammation is a risk factor for goblet cell hyperplasia, a hallmark of AECOPD, we evaluated goblet cells using AP-PAS staining (Fig. 4A–B). The rates of PAS staining in the control, model, LHQK-L, LHQK-M, LHQK-H, and Ambroxol groups were 0.07 ± 0.03%, 4.83 ± 0.58%, 3.51 ± 0.57%,1.64 ± 0.33%, 1.29 ± 0.29%, and 1.52 ± 0.27% (Fig. 4A, B), respectively. Notably, the number of PAS-positive cells was significantly greater in the model group than in the control group (*P* < 0.01), indicating goblet cell hyperplasia. Importantly, the number of goblet cells reduced effectively upon LHQK (LHQK-M and LHQK-H) and Ambroxol treatment (*P* < 0.01). The PAS-positive staining area was smaller in the Ambroxol group than in the LHQK-L group (*P* < 0.01), but the area showed no statistically significant difference compared to the areas in the LHQK-M and LHQK-H groups (*P* > 0.05).

**Fig. 5:**
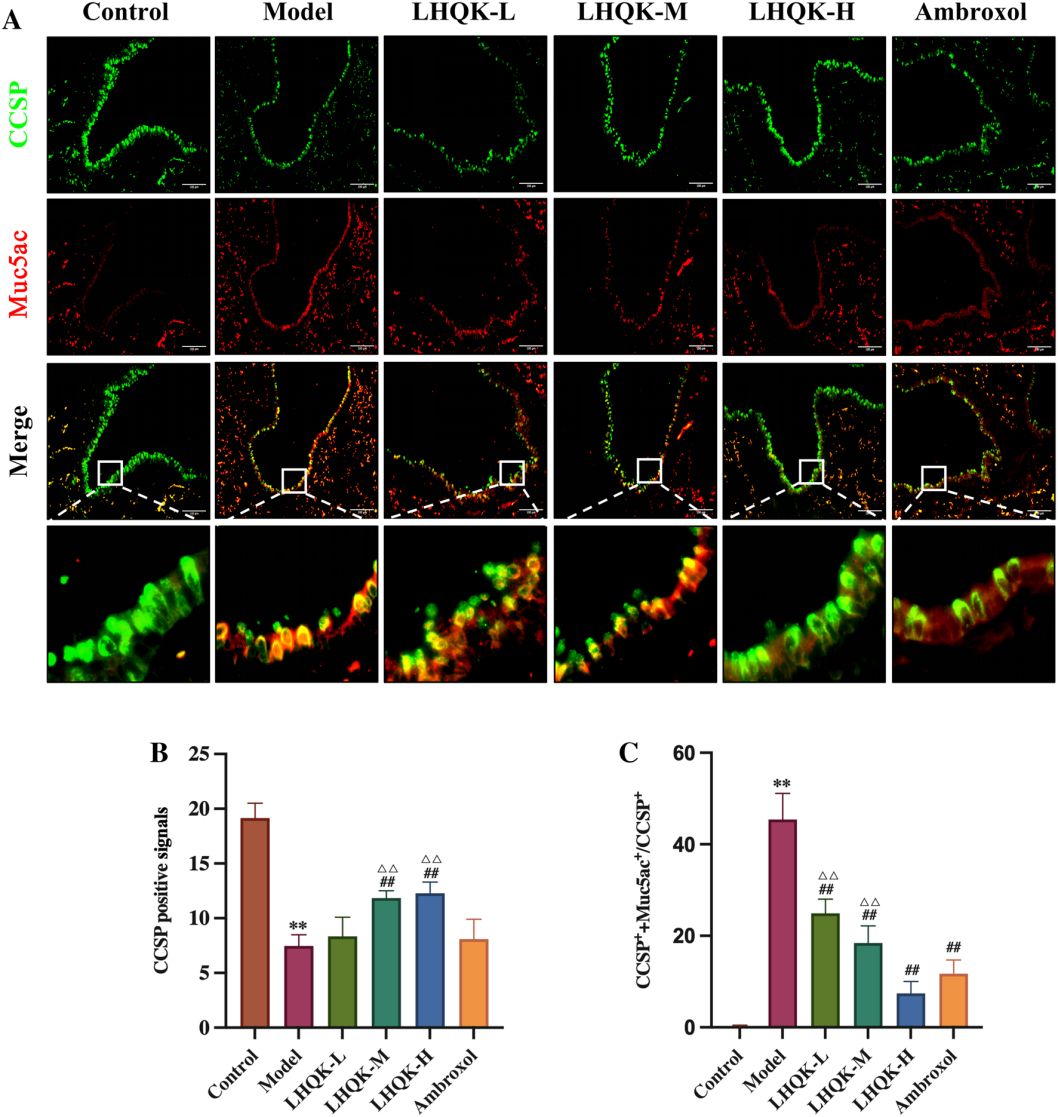
Effect of LHQK on the transdifferentiation of Club cells to goblet cells. **A** Immunofluorescence staining of CCSP (green) and Muc5ac (red) (confocal microscopy × 200) Bar: 100 μm. **B–C** Quantification of the positive signals of CCSP **B**. CCSP + /Muc5ac + double-positive signals in CCSP + cells **C** in lung tissues from rats (n = 3). The values are expressed as mean ± SD. One-way ANOVA was used for statistical analysis. ^**^P < 0.01 vs. the control group; ^##^*P* < 0.01 vs. the model group; ^∆∆^*P* < 0.01 vs. the Ambroxol group

Under injury stimulation, Club cells could transdifferentiate into goblet cells and contribute to goblet cell hyperplasia and mucin secretion [17]. Therefore, we next evaluated the transdifferentiation of Club cells to goblet cells (CCSP^+^ and Muc5ac^+^) using immunofluorescence staining with anti-CCSP and anti-Muc5ac antibodies (Fig. 5A). The CCSP^+^ signals in the model group were significantly weaker compared to those in the control group (*P* < 0.01) (Fig. 5B) but strengthened upon LHQK (LHQK-M, LHQK-H) and Ambroxol treatment (Fig. 5A, B). CCSP expression in the LHQK-M and LHQK-H groups was significantly greater than that in the Ambroxol group (*P* < 0.01). Interestingly, more CCSP^+^/Muc5ac^+^ double-positive cells were observed in the model group (*P* < 0.01), but the number of cells reduced in response to LHQK and Ambroxol treatment (*P* < 0.01) (Fig. 5A, C). This indicats the potential role of LHQK in inhibiting the transdifferentiation of Club cells into goblet cells.

### LHQK inhibited mucus hypersecretion and maintained mucus homeostasis

**Fig. 6:**
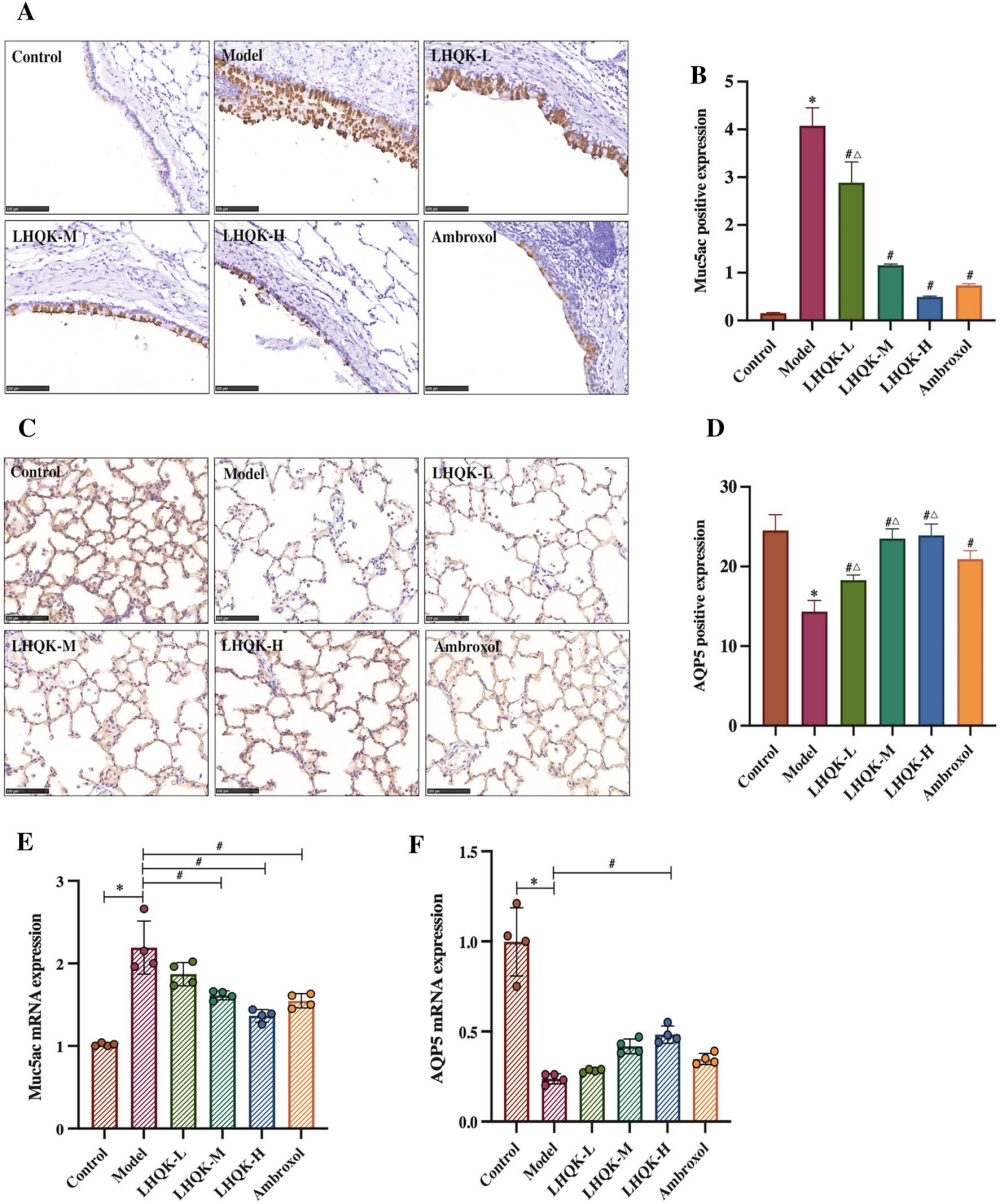
Effect of LHQK on Muc5ac hypersecretion and mucus homeostasis in lung tissues from rats. **A–B**. Immunohistochemical staining (magnification × 200) Bar: 100 μm. **A** and quantification of the protein levels of Muc5ac in lung tissues from rats (n = 5) **B. C–D** Immunohistochemical staining (magnification × 200) Bar: 100 μm. **C** and quantification of the protein levels of AQP5 in lung tissues from rats (n = 5) (D). **E–F** RT-qPCR analysis of the mRNA expression of Muc5ac and AQP5 in lung tissues from rats (n = 4). The values are expressed as mean ± SD. One-way ANOVA was used for statistical analysis. ^*^P < 0.05 and ^**^P < 0.01 vs. the control group; ^##^*P* < 0.01 and ^#^*P* < 0.05 vs. the model group; ^∆∆^*P* < 0.01 and ^∆^*P* < 0.05 vs. the Ambroxol group

In many obstructive pulmonary diseases, excessive secretion, especially Muc5ac, and imbalanced water-to-mucin ratios can lead to increased mucus viscosity and airway obstruction [14, 21]. To determine the role of LHQK on muc5ac secretion and mucus homeostasis, the protein and transcription levels of both Muc5ac and AQP5 were analyzed using immunohistochemistry (IHC) and RT-qPCR, respectively. Muc5ac staining showed upregulation of muc5ac in the model group (*P* < 0.05) and observable downregulation upon LHQK and Ambroxol treatment (*P* < 0.05) (Fig. 6A, B). In contrast, the level of AQP5, which was lower in the model group (*P* < 0.05), increased significantly (*P* < 0.05) upon LHQK and Ambroxol treatment (Fig. 6C, D). Consistent with this, Muc5ac transcription was considerably elevated and AQP5 transcription was considerably lower in the model groups compared to those in the control groups (*P* < 0.05), as shown by RT-qPCR (Fig. 6E, F). Compared with that in the model group, the transcription levels of Muc5ac in the LHQK-M, LHQK-H, and Ambroxol groups were significantly lower (*P* < 0.05). Additionally, the transcription level of AQP5 in the LHQK-H group was significantly higher (*P* < 0.05). Compared with that in the Ambroxol group, the AQP5 protein level was significantly lower in the LHQK-L group (*P* < 0.05) and considerably higher in the LHQK-M and LHQK-H groups (*P* < 0.05) (Fig. 6F).

**Fig. 7:**
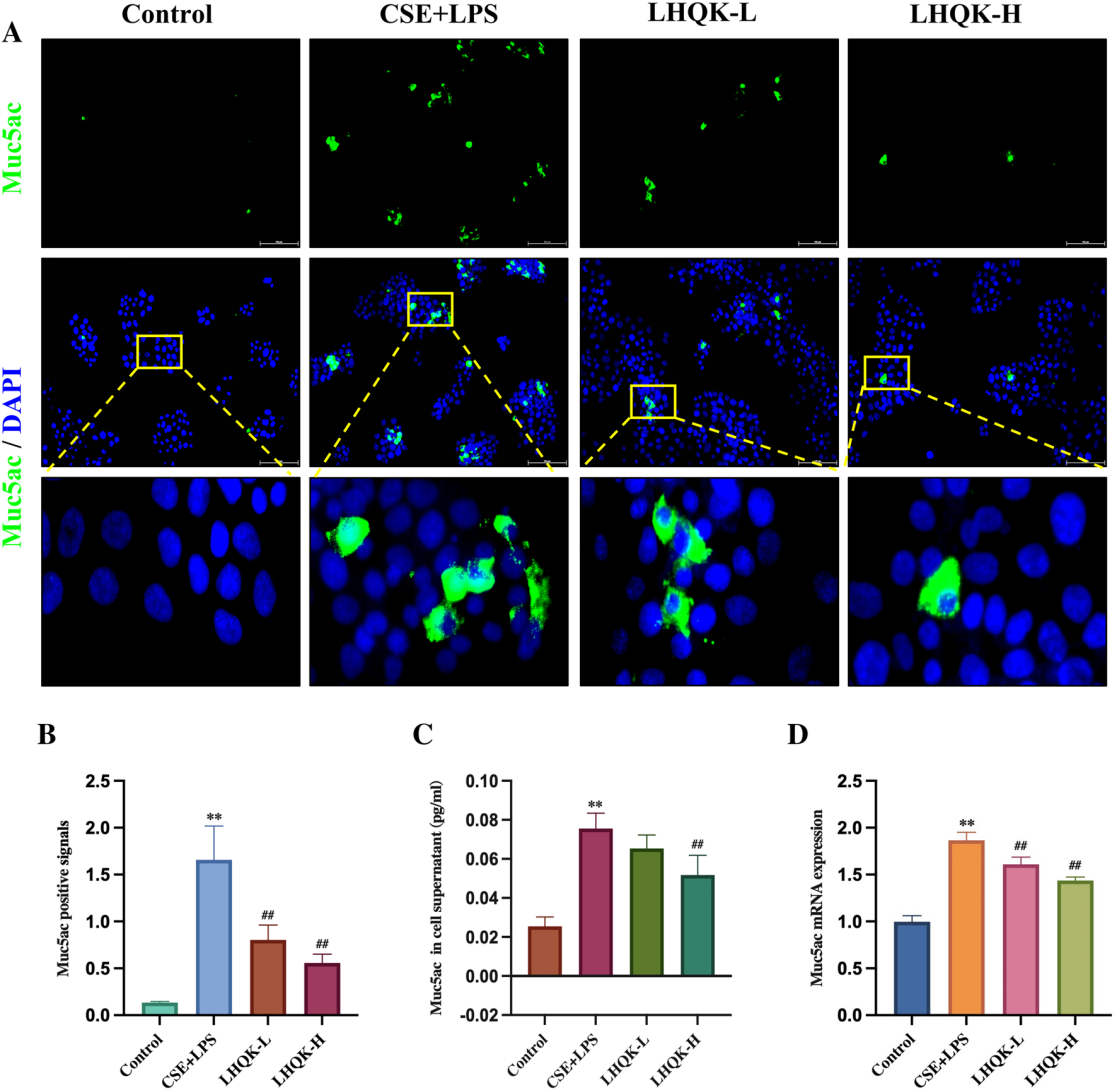
Effects of LHQK on the expression of MUC5AC in human airway epithelial cell NCI-H292. **A** Immunofluorescence staining with Muc5ac (magnification × 200) Bar: 100 μm. **B** Quantification of the positive signals of Muc5ac. **C **The expression of MUC5AC protein in cell supernatant (n = 6). **D** RT-qPCR analysis of the mRNA expression of Muc5ac in NCI-H292 cells (n = 3). The values are expressed as mean ± SD. One-way ANOVA was used for statistical analysis. **P < 0.01 vs. the control group; ^##^ *P* < 0.01 vs. the CSE + LPS group

Additionally, the impact of LHQK on the protein and mRNA levels of Muc5ac was investigated in an in vitro cellular model of mucus hypersecretion induced by CES and LPS exposure in the NCI-H292 human airway epithelial cell line. The protein levels of Muc5ac were assessed using immunofluorescence and ELISA with anti-Muc5ac antibody, while the transcription levels of Muc5ac was evaluated by RT-qPCR. Immunofluorescence analysis revealed a significant increase in the quantification of Muc5ac positive signals in the CS + LPS group compared to the control group (*P* < 0.01), whereas LHQK treatment effectively reduced Muc5ac positive signals (*P* < 0.01) (Fig. 7A, B). The ELISA results consistently demonstrated a significant increase in the protein level of Muc5ac in the CSE + LPS group compared to the control group (*P* < 0.01). However, this increase was found to be reduced upon LHQK treatment (Fig. 7C). Additionally, the RT-qPCR results indicated an upregulation of Muc5ac transcription in the CSE + LPS group, which was reduced by LHQK (*P* < 0.01) (Fig. 7D).

Collectively, the above in vivo and in vitro results demonstrate the therapeutic efficacy of LHQK in preventing muc5ac transcription and hypersecretion and regulating the water balance in mucus by controlling Muc5ac and AQP5 expression in AECOPD.

### Intervention mechanism of LHQK in airway mucus hypersecretion

**Fig. 8:**
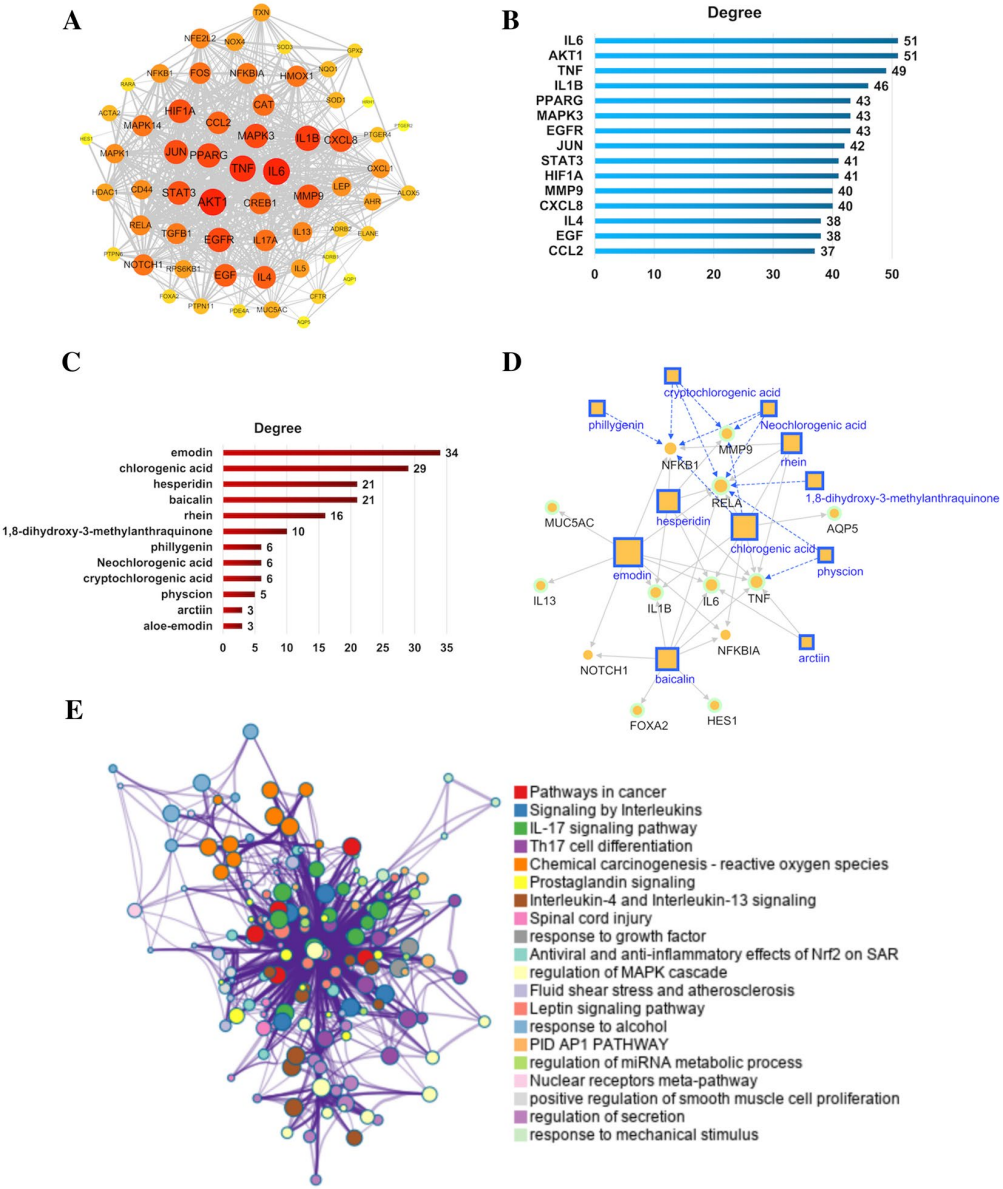
Network pharmacology analysis of potential targets regulated by the active ingredients of LHQK. **A** The PPI network of 61 targets. **B** Top 15 targets ranked by degree in the PPI network. **C** The ingredients ranked by degree in C-T networks. **D** The C-T network for 11 components and 13 targets associated with the transdifferentiation of Club cells to goblet cells, mucus secretion, and inflammatory factor secretion (24 nodes and 47 edges). Node size is proportional to the degree. The gray solid edge represents the known interactions, and the green dashed edge represents the predicted interactions. **E** BP and signaling pathway analysis of 61 potential targets regulated by LHQK. We selected a subset of representative terms from the full cluster and converted them into a network layout. More specifically, each term is represented by a circle node, where its size is proportional to the number of input genes under the term, and its color represents the cluster identity (i.e., nodes of the same color belong to the same cluster). Terms with a similarity score > 0.3 are linked by an edge (the thickness of the edge represents the similarity score). The network was visualized using Cytoscape with a “force-directed” layout and with edge bundled for clarity. One term from each cluster is selected to have the term description shown as a label

To explain the basis of LHQK action in ameliorating airway mucus hypersecretion, a PPI molecular network on the mechanism underlying airway mucus hypersecretion was constructed using 226 genes obtained from Gene Cards and CTD databases with the keyword "airway mucus hypersecretion" (Additional file 2: Table S1). By conducting an intersection analysis between the above targets and genes, 61 targets were identified as the mechanistic targets for the intervention effect of LHQK on airway mucus hypersecretion (Fig. 8A). Based on the node importance parameter, IL6, AKT1, TNF, IL-1β, PPARG, MAPK3, EGFR, JUN, STAT3, HIF1A, MMP9, and CXCL8, among others (Fig. 8B), were considered as hub nodes positioned at the core of the PPI molecular network. These nodes are closely related to the pathological mechanism of airway mucus hypersecretion. Of note, the expression of some of the targets, including IL6, TNF, IL-1β, and MMP9, was shown to be regulated by LHQK in our AECOPD model (Fig. 3A–D). Further, a compound-target (C-T) network between 14 components of LHQK(29) and 784 targets was constructed by integrating target data from multiple sources; this network comprised 1163 edges (Additional file 3: Table S2). Active components, such as emodin, chlorogenic acid, hesperidin, baicalin, rhein, and 1,8-dihydroxy-3-methylanthraquinone, were deduced to have a high degree in the compound-target (C-T) network (Additional file 4: Table S3 and Fig. 8C), which demonstrates their role in mediating the intervention effects of LHQK in airway mucus hypersecretion. Among the active ingredients, 11 components and 13 targets associated with the transdifferentiation of Club cells to goblet cells, mucus secretion, and inflammatory factors (24 nodes and 47 edges) are shown in the C-T network result (Fig. 8D).

Functionally grouped pathways and biological processes (BPs) were explored to interpret the mechanisms of action of LHQK in airway mucus hypersecretion (Fig. 8E and Additional file 5: Table S4). The key signaling pathways affected by LHQK in the treatment of airway mucus hypersecretion include interleukin signaling, IL-17 signaling, Th17 cell differentiation, prostaglandin signaling, interleukin-4 and interleukin-13 signaling, and antiviral and anti-inflammatory effects of Nrf2 on the SARS-CoV-2 pathway, among others. In addition, response to growth factor, regulation of the MAPK cascade, response to alcohol, regulation of miRNA metabolism, positive regulation of smooth muscle cell proliferation, regulation of secretion, and response to mechanical stimulus, among others, are the key biological processes involved in the therapeutic effect of LHQK on airway mucus hypersecretion.

**Fig. 9:**
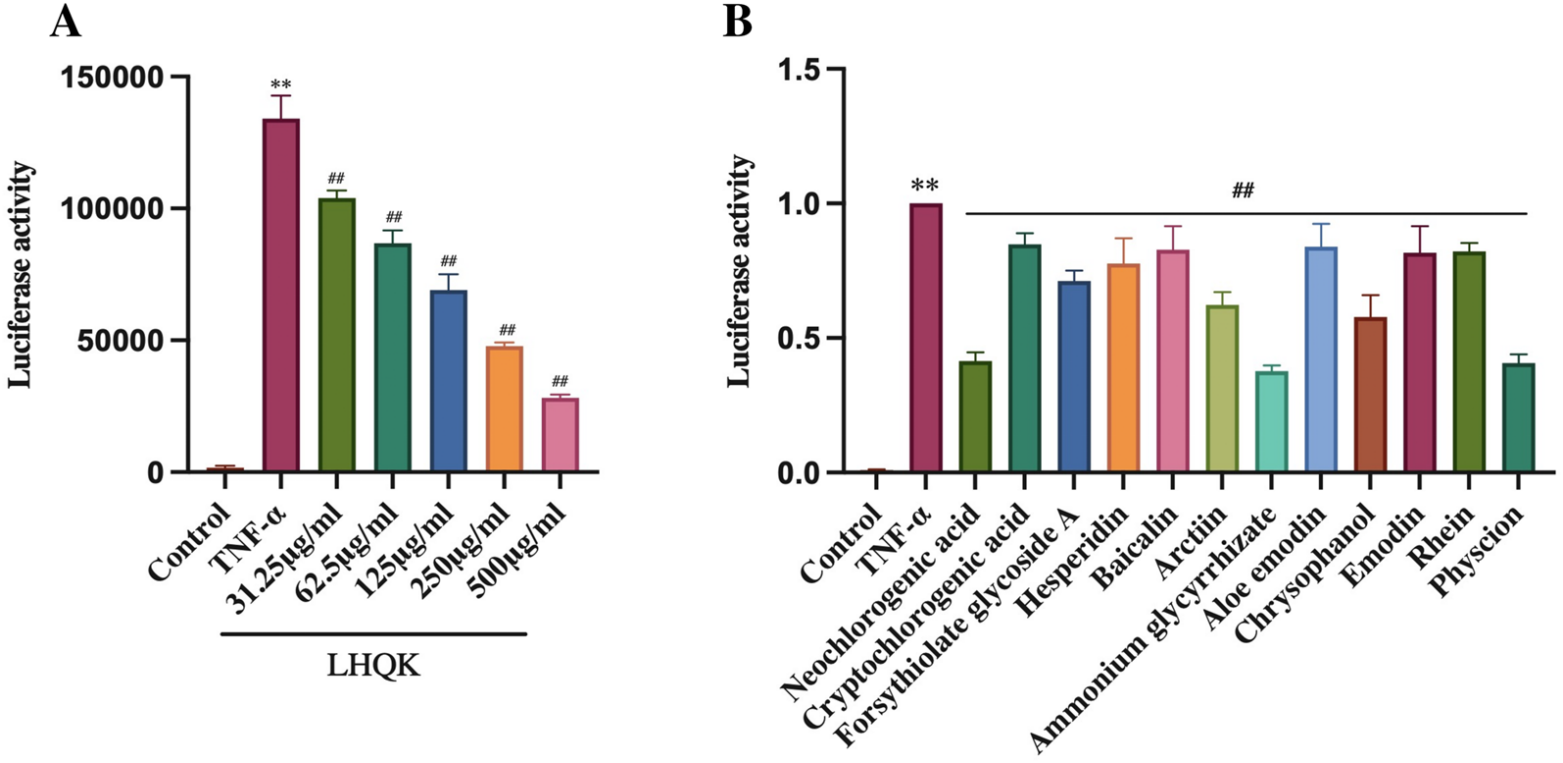
The inhibitory effect of LHQK and its active ingredients on NF-κB activity. **A** Inhibitory effect of LHQK on NF-κB activity. **B** Inhibitory effect of the active ingredients of LHQK on NF-κB activity. TNF-α was used to activate NF-κB. The dosages of the 12 ingredients were 100 μM for Neochlorogenic acid, Cryptochlorogenic acid, Forsythiolate glycoside **A**, Chrysophanol, Rhein; 10 μM for Hesperidin, Baicalin, Arctiin, Ammonium glycyrrhizate, Physcion, and 1 μM for Aloe emodin, Emodin. The values are expressed as mean ± SD. One-way ANOVA was used for statistical analysis. ^**^P < 0.01 vs. the control group; ^##^*P* < 0.01 vs. the TNF-α group

Given that NF-κB, which plays a central role in airway inflammation in COPD [51], was predicted as a target of active ingredients present in LHQK (Fig. 8B), we validated the inhibitory property of LHQK and its active ingredients on NF-κB using an NF-κB luciferase reporter system (Fig. 9). TNF-α was used to trigger NF-κB activation. LHQK dose-dependently inhibited NF-κB (Fig. 9A). Meanwhile, active ingredients, including neochlorogenic acid, cryptochlorogenic acid, forsythiolate glycoside A, hesperidin, baicalin, arctiin, ammonium glycyrrhizate, aleo-emodin, chrysophanol, emodin, rhein, and physcion, significantly inhibited NF-κB signaling (Fig. 9B). Collectively, these results confirm that the active ingredients of LHQK may suppress NF-κB signaling to regulate airway inflammation.

## Discussion

According to TCM theory, AECOPD is characterized by phlegm-heat obstruction in the lungs [52]. Airway mucus hypersecretion and inflammation are critical pathophysiological characteristics of AECOPD [12, 53]. Typically, AECOPD is triggered by bacterial or viral infections, or uninfected stimuli, which leads to inflammation and secondary mucus hypersecretion [54]. Unresolved mucus hypersecretion can lead to the formation of mucus plugs that block the airways and compromise the survival of patients with COPD. Therefore, interventions aimed at reducing excessive mucus accumulation may help reduce the exacerbation rate and lead to important health and economic benefits. In the present study, we successfully induced a rat AECOPD model with CS exposure and LPS instillation [26]. The model exhibited inflammatory cell infiltration and airway mucus accumulation. Further, we confirmed that LHQK can attenuate pulmonary structure abnormality and airway inflammation, possibly through MMP-9 regulation and anti-inflammatory effects, respectively. The patterns of Muc5ac and AQP5 expression indicate the role of LHQK in regulating mucin secretion and maintaining mucus homeostasis. Mechanistically, LHQK may inhibit goblet cell hyperplasia by controlling the transdifferentiation of Club cells into goblet cells. Furthermore, we used network pharmacology analysis to predict that the active ingredients of LHQK may regulate airway mucus hypersecretion and validated the effect of LHQK and its active ingredients on NF-κB signaling inhibition using a luciferase reporter system.

Goblet cells are specialized cells present in the respiratory tract that are responsible for producing and secreting mucus. In AECOPD, the excessive production of inflammatory mediators, such as cytokines and growth factors, leads to an increase in goblet cell hyperplasia and differentiation, which play a significant role in mucin secretion. Our findings indicate that LHQK significantly suppressed airway mucus hypersecretion in AECOPD rats as well as in the NCI-H292 human airway epithelial cell line in vitro. Accumulating evidence confirms that the transdifferentiation of Club cells to goblet cells may contribute to goblet cell hyperplasia after lung injury [17, 18]. Therefore, therapeutic strategies targeting goblet cell differentiation, which is not considered a major pharmacological target at present, may be useful to prevent COPD exacerbation. Here, we observed that LHQK could effectively reduce the number of PAS-positive goblet cells and CCSP^+^/Muc5ac^+^ double-positive cells in rat AECOPD, suggesting that LHQK may prevent goblet cell hyperplasia by suppressing the transdifferentiation of Club cells to goblet cells. However, whether the regulatory effects of LHQK on Club cell transdifferentiation are directly or indirectly mediated through inflammatory control should be further investigated.

In addition to the high level of mucus secretion, the difficulty in lung exudate discharging is closely related to the increase in mucus viscosity. The imbalance in the water-mucin ratio is also a decisive factor for the increase in airway mucus viscosity [14]. Normal airway mucus mostly contains water (95%) along with mucin glycoproteins (3%) [21]. The proportion of water and the quality of airway mucus is regulated by water channel proteins, also known as aquaporins (AQPs), expressed in the airway cells. AQP5 is an important AQP and is primarily expressed by type I alveolar epithelial cells (AT1) [22]. AQP5 expression was shown to be negatively correlated with Muc5ac expression, possibly through intracellular signaling pathways [55]. In our study, LHQK significantly increased AQP5 expression in the rat AECOPD model, indicating the regulatory function of LHQK in airway mucus homeostasis. Additionally, compared to Ambroxol, LHQK (in the LHQK-M and LHQK-H groups) exerted a superior effect in maintaining the protein levels of AQP5 in the AECOPD model.

LHQK is composed of 15 herbs and is made from two classical TCM prescriptions: Maxing Shigan decoction and Qingjin Huatan decoction [56, 57]^.^ The advantage of TCM formulations lies in their ability to target different pathways and play integrative roles in disease treatment. In our study, LHQK, which had been shown to exhibit broad-spectrum antibacterial and antiviral effects, was found to inhibit mucus hypersecretion in CS + LPS-induced AECOPD. To gain insights into the relationship between active ingredients and biological activities, we performed network pharmacology analysis using the ingredients identified in the published LHQK UPLC/MS data [29], along with genes and signaling pathways related to airway mucus hypersecretion. Active ingredients, including emodin, chlorogenic acid, hesperidin, baicalin, rhein, and 1,8-dihydroxy-3-methylanthraquinone, were predicted with high degree in regulating signaling pathways involved in responses to inflammation, responses to growth factors, regulation of secretion, and so on. Additionally, analysis conducted using the NF-κB luciferase reporter system confirmed that LHQK and its active ingredients may directly regulate inflammation through the NF-κB related signaling pathway. These findings provide a pharmacological basis for understanding the effects of LHQK.

## Conclusion

In conclusion, our findings confirm that LHQK can relieve airway obstruction symptoms by preventing inflammatory cell infiltration, inhibiting goblet cell hyperplasia, ameliorating airway mucus hypersecretion, and maintaining liquid homeostasis in AECOPD. Despite the existence of more questions on this topic, these results will facilitate the clinical application of LHQK for the treatment of AECOPD.

### Supplementary Information

**Additional file 1: Figure S1.** 24 h cell viability of LHQK and 12 active ingredients in LHQK.

**Additional file 2: Table S1.** 233 genes closely related to the airway mucus hypersecretion.

**Additional file 3: Table S2.** Component target profiles from multiple sources for compound-target (C-T) network construction.

**Additional file 4: Table S3.** Compound-target (C-T) network for 61 overlap targets.

**Additional file 5: Table S4.** Pathways and biological process (BP) enrichment analysis of 61 potential targets regulated by LHQK.

## Data Availability

The data used to support the fndings of this study are available from the corresponding author upon request.
