# Supplementary material for: Therapeutic potential of Lianhua Qingke in airway mucus hypersecretion of acute exacerbation of chronic obstructive pulmonary disease
Source: Chin Med. 2023 Nov 3;18:145. doi: 10.1186/s13020-023-00851-4 (PMC10623880; doi:10.1186/s13020-023-00851-4)
Supplement: Supplementary file 1 — Additional file 1: Figure S1. 24 h cell viability of LHQK and 12 active ingredients in LHQK. [file 13020_2023_851_MOESM1_ESM.pdf]

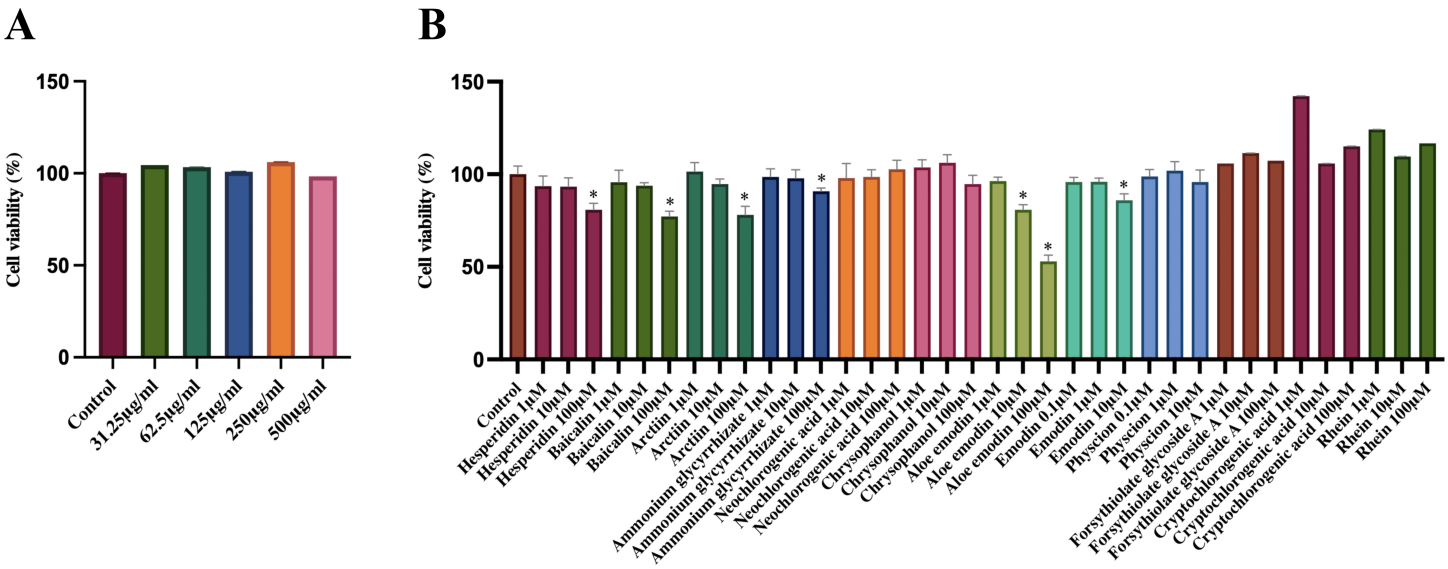

Figure S1. 24-hour cell viability of LHQK and 12 active ingredients in LHQK. **(A)** Cell viability of LHQK at five different doses. **(B)** Cell viability of 12 active ingredients at three different doses. The values are expressed as mean ± SD. One-way ANOVA was used for statistical analysis. *$P < 0.05$ vs. the control group.
